# The Switch From Paliperidone Long-Acting Injectable 1- to 3-Monthly: Clinical Pharmacokinetic Evaluation in Patients With Schizophrenia (Preliminary Data)

**DOI:** 10.1097/JCP.0000000000001484

**Published:** 2021-11-29

**Authors:** Massimo Carlo Mauri, Gemma Franco, Alessandro Minutillo, Silvia Paletta, Chiara Di Pace, Alessandra Reggiori, Sara Baldelli, Dario Cattaneo

**Affiliations:** From the ∗ Department of Neurosciences and Mental Health; † Clinical Psychopharmacology Unit, Fondazione IRCCS Ca' Granda Ospedale Maggiore Policlinico; ‡ Clinical Pharmacology Unit, Luigi Sacco University Hospital, Milan, Italy.

**Keywords:** schizophrenia, atypical antipsychotics, psychosis, long-acting antipsychotics, paliperidone, therapeutic drug monitoring, plasma levels of atypical antipsychotics, pharmacokinetics, plasma concentrations

## Abstract

**Purpose/Background:**

The aim of the study was a preliminary evaluation of the maintenance of clinical efficacy and tolerability of paliperidone palmitate in patients with schizophrenia during the transition phase from 1-monthly paliperidone palmitate formulation (PP1M) to PP3M, with the evaluation of plasma levels of the drug.

**Methods/Procedures:**

A prospective observational study was conducted for 13 months involving 22 outpatients, aged 18 to 66 years and clinically stabilized. Patients were affected by schizophrenia according to *Diagnostic and Statistical Manual of Mental Disorders*, *Fifth Edition*, criteria. For each patient, clinical assessment, safety and tolerability, and drug plasma level determination were performed. Clinical efficacy was assessed by Brief Psychiatric Rating Scale, Positive and Negative Symptom Scale, and Hamilton Rating Scale for Depression. During the first 4 months of the study, once-monthly paliperidone palmitate was administered, and then during the following 9 months, the 3-monthly formulation was administered.

**Findings/Results:**

The time course of the Brief Psychiatric Rating Scale total scores showed a statistically significant (*P* = 0.006) improvement from T_0_ to T_8_; Positive and Negative Symptom Scale scores showed a similar time course, with a statistically significant (*P* = 0.0016) reduction of the mean total score; Hamilton Rating Scale for Depression mean scores showed a statistically significant (*P* = 0.003) reduction with substantial maintenance of clinical stabilization of the patients. Only 1 patient dropped out after the first PP3M injection.

**Implications/Conclusions:**

Our preliminary data currently confirm the maintenance of clinical stability shifting from PP1M to PP3M.

Poor adherence to antipsychotic treatment is one of the major issues in the management of patients with schizophrenia, resulting in an increased risk of relapses and rehospitalization, poorer long-term clinical outcome, and higher costs for the mental health system and society. In this framework, long-acting injectable (LAI) formulations of antipsychotic drugs were introduced in the 60s, with the aim to improve compliance, allowing the medication to be administered biweekly or monthly. Currently, LAI formulations have been proved to provide sustained therapeutic plasma concentrations for several weeks reducing relapse rates and providing long-term clinical benefits. Moreover, depot formulations allow to avoid first-pass hepatic metabolism, increasing drug bioavailability and possibly leading to lower total dosage administration with fewer dose-related adverse effects.^1–5^

This therapeutic strategy represents one of the best options for patients and their caregivers to improve the long-term outcome of schizophrenia by reducing the high rates of relapses and rehospitalizations due to treatment discontinuation.^6^

In a long-term, real-world study on 29,823 patients affected by psychosis, Tiihonen et al^7^ (2017) investigated the effectiveness of antipsychotic treatments in terms of risk of rehospitalization and treatment failure, defined as psychiatric rehospitalization, suicide attempt, discontinuation/switch to other medication, or death. Results showed LAI antipsychotic medications to be the pharmacologic treatments with the highest rates of relapse prevention in schizophrenia. The risk of rehospitalization was 22% lower during LAI treatments compared with equivalent oral formulations, even in patients at their first episode.^7^

The use of LAI formulations of antipsychotics has been subjected to stigma over the past years, and it still is in some contexts, where it is often considered the last treatment option for chronic patients. Current evidence has changed the perspective about the use of depot formulations as a first option: they can be considered first-choice drugs in first-episode psychosis as well, with proven efficacy and good tolerability. Thus, using LAI as a first option at the onset of the disease in schizophrenia can have a beneficial impact on treatment outcome preventing functional deterioration linked to relapses.^8^

Paliperidone palmitate (PLP) is a LAI monthly formulation of paliperidone (PP1M), the primary active metabolite of risperidone, approved by the Food and Drug Administration in 2009 for acute and maintenance treatment of schizophrenia and schizoaffective disorder. Moreover, it is the only LAI prescriptible requiring neither a previous oral administration nor any initial oral supplementation. Although the marketer suggests that there is no need for oral supplementation, clinicians frequently revert to it and other authors recommend it as well.^9^

A recently developed 3-monthly formulation (PP3M) uses a NanoCrystal1 technology similar to that of PP1M, but with increased particle size, providing an extended sustained release of paliperidone and permitting a significantly extended dosing interval (4 doses per year).^10^

Multiple studies have demonstrated the safety and efficacy of PP1M (25, 50, 75, 100, or 150 mg eq; deltoid or gluteal injections) in patients with schizophrenia. Short-term studies have reported that stable plasma levels (PLs) of PP1M improved clinical outcomes, notably leading to the amelioration of schizophrenic clinical symptoms, with an efficacy and safety profile similar to that of flexibly dosed risperidone long-acting injection.^11–16^

In a 13-week, multicenter, randomized, double-blind study on 514 patients, Nasrallah et al^17^ (2010) investigated the efficacy, safety, and tolerability of various doses of PP1M compared with placebo. They found a significantly improved change in PANSS total scores compared with the placebo group.

Furthermore, during long-term studies, PP1M was shown to reduce the probability of disease relapse, improve patient functioning, and reduce hospitalization.^18,19^

In a 52-week, open-label study following a double-blind phase conducted on 388 patients, Gopal et al^12^ (2011) observed an improvement of schizophrenic symptoms in patients treated with PP1M instead of placebo. All clinical assessments showed additional improvement during the open-label phase for patients on continued treatment with paliperidone palmitate. As expected, the most significant improvement in PANSS total scores was observed in patients who were on placebo during the double-blind phase and who received PP1M upon entering the open-label phase. All the patients on uninterrupted paliperidone palmitate treatment had the best PANSS scores at all time points, and most patients showed an improvement in personal and social functioning as well.^12^

On the other hand, some authors have pointed out the critical issues linked to the clinical use of PP1M. According to Schoretsanitis et al^9^ (2018), data on steady-state therapeutic drug monitoring (TDM) of PP1M is very limited, and there is an urgent need to investigate real-world PP1M *t*_1/2_ because it is required to estimate steady state. The PP1M product monograph reports a median “apparent” *t*_1/2_ ranging from 25 to 49 days, over the dosage strengths of 25–150 mg eq. Currently, clinicians should consider steady state to be reached no earlier than after 9 injections of PP1M (8 months), but it may be longer for obese patients and possibly for female patients.^9^

Consistent with the previous investigations, the main limitation in the administration of paliperidone (in any of its available formulations) could be represented by the onset of hyperprolactinemia (especially in women) and mild parkinsonism.^20^ Furthermore, the associated symptoms of hyperprolactinemia, like sexual dysfunction, are the most manifest significant limitation from the patient's perspective.

In 2015 in the United States, and in 2016 in Italy, paliperidone palmitate was introduced in its PP3M for the maintenance treatment of schizophrenia in patients who had been previously treated with PP1M for at least 4 months. In the United States, it was also allowed for the treatment of schizoaffective disorder in adults, as monotherapy and as an adjunctive treatment to mood stabilizers or antidepressants.^21^

Unlike the case of PP1M, in which it is highly recommended to start with a gluteal injection, the first injection of PP3M can be both deltoid and gluteal, taking some pharmacokinetic differences into consideration.^10^

The dosage is closely connected to that of PP1M previously prescribed to the patient and should be 3.5 times the dose of the monthly injections of paliperidone that the patient was previously receiving.^10^

Recent studies evaluated efficacy, safety, and tolerability of PP3M, demonstrating equal safety and efficacy in PP3M and PP1M. The 3-monthly paliperidone palmitate injection might be a useful treatment option for the maintenance treatment of patients with schizophrenia, particularly for those who would prefer, or may benefit from, longer dosing intervals.^22^ In a randomized, multicenter trial conducted from 2012 to 2014 on 283 patients with schizophrenia, Berwaerts et al^23^ (2015) demonstrated that 93% of patients who were adequately stabilized with PP1M for at least 4 months and subsequently treated with PP3M did not experience a significant return of schizophrenia symptoms. Three-monthly paliperidone palmitate formulation significantly delayed time to relapse compared with placebo.^23^ A second multicenter, double-blind, long-term study was designed by Savitz et al^24^ (2016) to test the noninferiority of PP3M versus PP1M on 1016 patients with schizophrenia. Three-monthly paliperidone palmitate formulation showed to be noninferior to PP1M, with similar relapse rates in both groups (8% in PP3M group vs 9% in PP1M group).^24^

Concerning pharmacokinetics, due to the extremely long half-life of PP3M, it is recommended to switch treatment in patients who have received at least 4 months of PP1M, to assess individual tolerability. Because no clinically meaningful pharmacokinetic data are available for PP3M, other than a single-dose trial,^1^ it is impossible to define steady-state conditions for PP3M: it is very likely that it should take more than 1 year, and it would not be surprising if future studies reported detectable PP3M blood levels for several years after PP3M discontinuation. It has been suggested that PP3M remains active for 1 1/2 years.^25^ Moreover, Lee et al^26^ proposed that PP3M steady state is achieved in approximately 1 1/2 years for deltoid injections and almost 2 years for gluteal injections. Clinicians should be recommended using repeated long-term TDM in any patient switched from PP1M to PP3M.^9^ Moreover, it should be considered that the half-lives of second-generation LAI antipsychotics reported in product monographs were often determined using small sample sizes, and times to steady state were determined via visual inspection of plasma concentration-time curves with not quite conservative estimates. Unfortunately, the pharmacokinetic information found in the product monographs of LAI antipsychotics is of limited help to clinicians who wish to optimize their effectiveness and tolerability. Relying on product monograph values may result in an overshooting of the dosage and the delayed onset of adverse events.^26^

## PURPOSE OF THE STUDY

The aim of the study was to evaluate the maintenance of clinical efficacy and tolerability of paliperidone palmitate in patients with schizophrenia during the transition phase from PP1M to 3-PP3M. We also investigated PLs variations of paliperidone during the switch from PP1M to PP3M.

## METHODS AND PROCEDURES

This is a prospective observational study conducted in the psychopharmacology unit of the Fondazione IRCCS Ca' Granda Ospedale Maggiore Policlinico in Milan, Italy.

Adult patients with a diagnosis of schizophrenia were recruited since 2017, and the observation is still ongoing. The administration of paliperidone palmitate was proposed by clinicians and discussed with patients and their caregivers. Efficacy, safety, and tolerability of the medication were illustrated.

The protocol was approved by the local ethical committee, and informed consent was obtained from the patients or their relatives after the study was fully described.

The study involved 22 outpatients, aged 18 to 66 years and clinically stabilized. Patients were affected by Schizophrenia according to *Diagnostic and Statistical Manual of Mental Disorders, Fifth Edition,* criteria, diagnosed by structured clinical interview (“Structured Clinical Interview for *Diagnostic and Statistical Manual of Mental Disorders*, *Fifth Edition*,” American Psychiatric Association, 2015).

The demographic and clinical characteristics of the patients are shown in Table 1.

**TABLE 1:** Demographic and Clinical Characteristics of the Patients Studied

Patients (N = 22; 10 F, 12 M)	Mean	SD	Min	Max
Age, y	46.91	12.13	20	66
Age of onset, y	24.82	6.88	17	36
Disease duration, y	21.27	10.87	2	41
DUI, mo	19.64	19.09	0	68
Total no. hospitalizations	3.36	3.49	0	15
No. PLP hospitalizations	1	0	0	1
PP3M posology, mg	317.31	105.29	175	525

DUI, duration of untreated illness; F, female; M, male; Max, maximum value; Min, minimum value.

Major exclusion criteria were: substance use disorders (various severities) within 30 days before screening, neurocognitive disorders (various severities), significant risk of suicidal behavior, any unstable or significant medical or neurological illness, relapse and/or hospitalization, nonadherence to prescribed therapies, renal impairment, age of older than 66 years, pregnancy, breastfeeding status, and concomitant therapy with any potential metabolic inducer. Patients with a history of intolerability, hypersensitivity, or lack of response to risperidone or paliperidone were also excluded from the study. No other concomitant treatments have been provided to the patients included in this study, apart from benzodiazepines in case of dire necessity.

For each patient, the following demographic and clinical characteristics were collected: age, sex, diagnosis, duration of illness, duration of untreated illness, age at first treatment, duration of previous therapy with PP1M, total number of previous hospitalizations, and number of hospitalizations during treatment with paliperidone.

### Clinical Assessment and Treatment Administration

Clinical assessment and drug administration were carried out on an outpatient procedure to allow clinical observation and rapid access to appropriate medical care in case of adverse events. Patients underwent psychiatric visit, drug administration, and venous sampling on every access. Patients and their caregivers were invited to pay attention and report signs and symptoms of treatment-emergent adverse events on the administration day and on the following days and to refer to the clinician in case of necessity.

Each injection was administered by a trained nurse after shaking the syringe vigorously for at least 15 seconds in the 5 minutes before the administration to ensure a homogeneous suspension.

**FIGURE 1:**
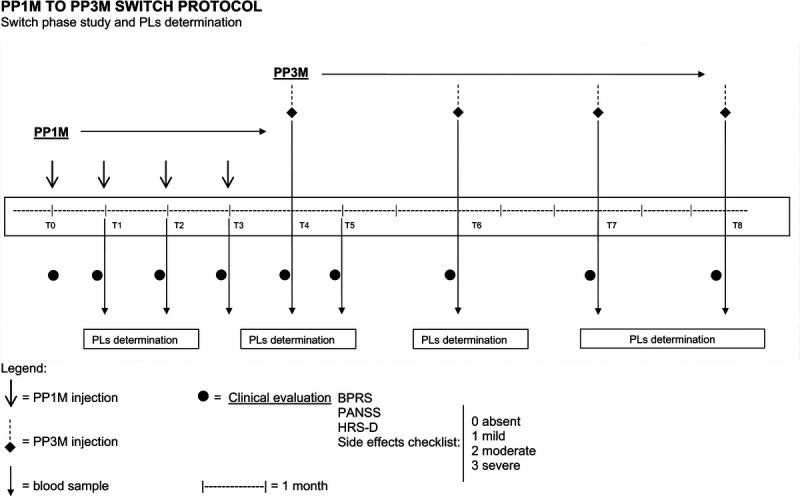
Protocol of the study.

For each patient, the clinical assessments were performed at T_0_ (time of the first PP1M administration) and every 28 days up to T_4_ (time of the switch to PP3M), then after 28 days (T_5_) after the first PP3M injection, then after 2 months (T_6_), and finally every 3 months until T_8_ (9 months of PP3M administration). The scheme is reported in Figure 1.

**FIGURE 2:**
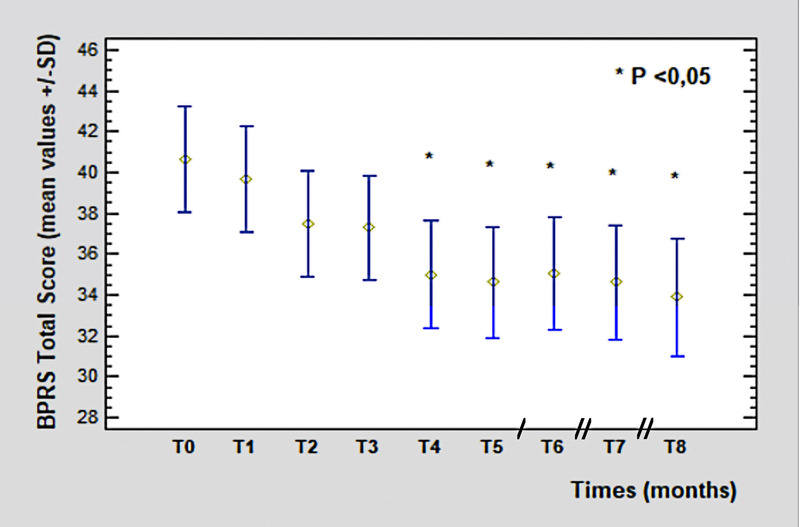
The BPRS total score from T_0_ (40.62 ± 1.55 SD) to T_8_ (33.88 ± 2.72 SD, *P* = 0.006). Legend: / = 2 months; // = 3 months. Color image is available online only at www.psychopharmacology.com.

**FIGURE 3:**
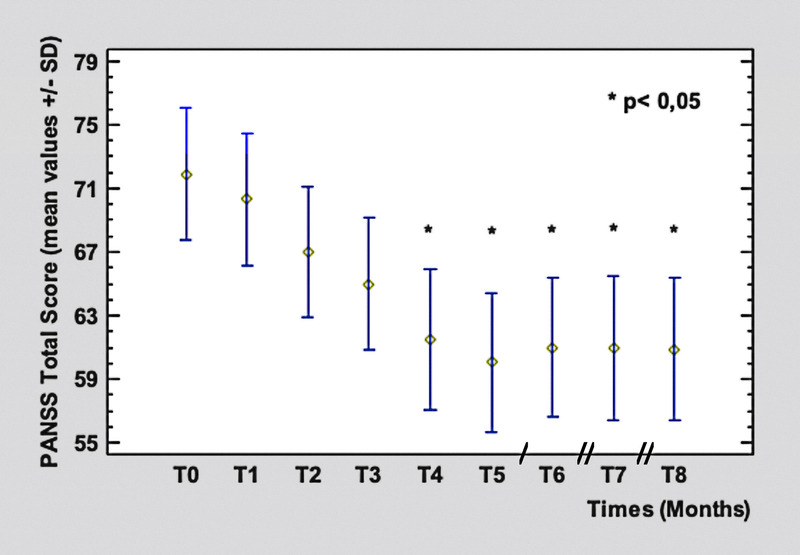
The PANSS total score from T_0_ (71.9 ± 3.9 SD) to T_8_ (60.88 ± 4.7 SD, *P* = 0.0016). Legend: / = 2 months; // = 3 months. Color image is available online only at www.psychopharmacology.com.

**FIGURE 4:**
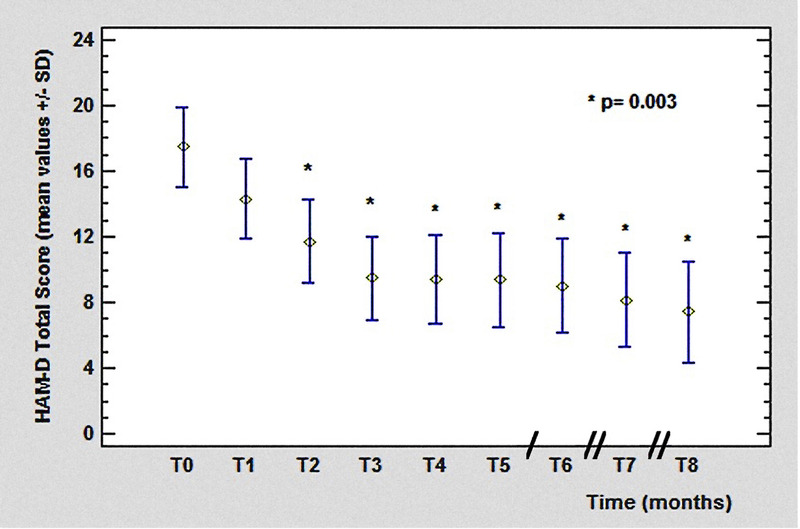
The HAM-D total score from T_0_ (17.45 ± 7.38 SD) to T_8_ (7.42 ± 5.97 SD, *P* = 0.003). Legend: / = 2 months; // = 3 months. Color image is available online only at www.psychopharmacology.com.

Clinical efficacy was assessed by Brief Psychiatric Rating Scale (BPRS),^27^ Positive and Negative Syndrome Scale (PANSS),^28^ and Hamilton Rating Scale for Depression (HAM-D21; Figs. 2–4).^29^ For BPRS, the following cluster of symptoms were also determined to get a more specific psychopathological evaluation and to better analyze the clinical course:

- Anxiety-depression (ANDP) including items n. 1–2–5–9;
- Anergy (ANER) including items n. 3–13–16–18;
- Ideation disorder (IDEA) including items n. 4–8–12–15;
- Activity (ACTV) including items n. 6–7–17;
- Hostility-suspiciousness (HOST) including items n. 10–11–14.

All clinical assessments were carried out by the same qualified examiners, previously trained for the use of the assessment scales and blinded about the patient's therapy.

Adverse effects were assessed at T_0_ and at every following access, through patient reports or clinical observation, and listed in the database.

Safety and tolerability were evaluated by body weight measurement, 12-lead electrocardiogram, and blood tests (in the correspondence of every plasma level determination), such as total cholesterol (CHO), low density lipoprotein, high density lipoprotein, triglycerides, glucose, aspartate aminotransferase, alanine aminotransferase, and γ-glutamyl transpeptidase (GGT).

Blood pressure and heart rate were measured before and after every injection, for a total of 2 measurements each time.

From T_0_ to T_4_, PP1M was administered, and from T_5_ to T_8_, the patients received the PP3M, following the therapeutic scheme reported in Figure 1.

All the patients had been previously treated with once-monthly injections of paliperidone palmitate for at least 4 months to stabilize the dosage and reach the steady state. The switch to the 3-monthly paliperidone palmitate formulation was proposed by clinicians on the basis of clinical judgment or requested by patients who wished to switch to a less frequent dosing schedule. Other oral antipsychotics were discontinued at the moment of the first injection of PP1M. All patients had not been treated with other long-acting antipsychotics for at least 12 months before entering the study.

No other drugs were administered apart from benzodiazepines in case of dire necessity.

### Plasma Levels Determination

Blood sampling to measure the PLs of the drug was carried out in the morning before the injection of the antipsychotic at T_1_, T_2_, T_3_, T_4_, T_5_ (28 days after the first PP3M injection), T_6_, T_7_, and T_8_ (at the fourth injection of PP3M; Fig. 1).

After blood drawing, the samples were analyzed using liquid chromatography/tandem mass spectrometry method at the centralized pharmacokinetics laboratory of the Unit of Clinical Pharmacology of the Azienda Socio Sanitaria Territoriale Fatebenefratelli Sacco University Hospital (Milano, Italy).

As described elsewhere, the plasmatic concentrations of paliperidone were analyzed by liquid chromatography/tandem mass spectrometry method (liquid chromatography-tandem mass spectrometry) by the same laboratory.^30^

### Statistical Analysis

The statistical analysis of the data was carried out through the Statgraphics software (Statpoint, Inc, http://www.statgraphics.com), version 2018. It included descriptive analyses, χ^2^ test, analysis of variance, multifactor analysis of variance (Tukey test), and analysis of linear regression.

## RESULTS

### Clinical Efficacy

Only 1 patient dropped out of the study because of relapse and hospitalization after the first injection of PP3M, with subsequent therapy modification. The characteristics of the patient at the time of drop out were: BPRS = 61, PANSS = 88, injection dosage = 175 mg, and PLP PLs = 13 ng/mL.

The time course of the BPRS total scores showed a statistically significant (*P* = 0.006) improvement from T_0_ (40.62 ± 1.55 SD) to T_8_ (33.88 ± 2.72 SD), starting from T_4_ (corresponding to the switch to PP3M), remaining constant in the following measurements.

Regarding BPRS clusters, the HOST cluster and ACTV cluster showed a significant improvement (T_0_: 7.9 vs T_8_: 4.25, *P* = 0.0009; T_0_: 6.8 vs T_8_: 5.25, *P* = 0.006, respectively). No significant variations were observed for the THOT, ANDP, and ANER clusters.

The PANSS scores showed a similar trend to those of BPRS, with a statistically significant (*P* = 0.0016) reduction of mean total score from T_0_ (71.9 ± 3.9 SD) to T_8_ (60.88 ± 4.7 SD). The improvement started from T_4_ and maintained constant values in the following months.

General psychopathology subscale mean scores were significantly reduced from T_0_ (38.1 ± SD) to T_8_ (23.5 ± SD, *P* = 0.0005) as well as positive symptoms subscale, with an improvement from T_0_ (16.4 ± 2.83 SD) to T_8_ (9.87 ± 2.75 SD, *P* = 0.002). The reduction observed for the negative symptoms' subscale scores (T_0_: 20.85 ± 6.87 SD vs T_8_: 18.35 ± 5.03 SD) was not significant.

The HAM-D rating scale mean scores showed a reduction from T_0_ (17.45 ± 7.38 SD) to T_8_ (7.42 ± 5.97 SD), statistically significant (*P* = 0.003) starting from T_2_ until the end of the study.

### Safety and Tolerability

**FIGURE 5:**
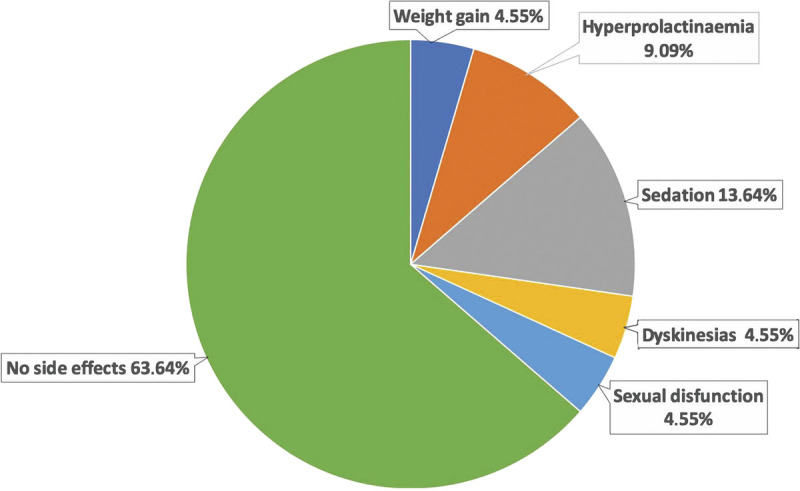
Adverse effects. Color image is available online only at www.psychopharmacology.com.

The adverse effects reported are shown in Figure 5.

No adverse effects were found in 63% of patients. Body weight showed a mean percentage of variation from the start to the end of the study of 4.2%, weight gain greater than 7% was reported in 4% of patients, increased prolactin levels in 9% (without related symptoms), dyskinesia in 4%, sexual dysfunction in 4%, and sedation in 14% during the first day after the injection. Blood GLU levels determined during the study varied from 81.05 ± 8.66 mg/dL (T_0_) to 89.37 ± 12.73 mg/dL (T_8_); total CHO values ranged from 183.47 ± 25.34 mg/dL (T_0_) to 197 ± 26.91 mg/dL (T_8_); triglyceride levels ranged from 126.94 ± 60.95 mg/dL (T_0_) to 148.9 ± 42.22 mg/dL (T_8_); GGT values varied from 20.52 ± 10.26 U/L (T_0_) to 21.14 ± 15.01 U/L (T_8_). No significant variations were reported for all these parameters throughout the study. No clinically relevant changes in vital parameters (pulse oximetry, blood pressure, heart rate, body temperature) and in electrocardiography were detected throughout the study. The local tolerability in the injection site was good: only 3 patients reported pain at the injection site, resolved in few hours, and only during the first injections. No differences were detected, in terms of local pain at the injection site, between PP1M and PP3M.

### Plasma Levels

Plasma levels varied from a minimum of 16.40 ng/mL to a maximum of 30.22 ng/mL for PP1M and from a minimum of 19.09 ng/mL to a maximum of 35.01 ng/mL for PP3M. The mean values at T_4_ and T_5_ (28 days after the first PP3M injection) during the switch from PP1M to PP3M are shown in Table 2.

**TABLE 2:** Mean of Values and % of Patients Within Ranges <10, <20, or >20 ng/mL at T_4_ and T_5_, During the Switch From PP1M to PP3M

	T_4_	T_5_
PLs mean, ng/mL	32.84	23.56
PLs SD, ng/mL	15.21	11.98
PLs min, ng/mL	6.9	6.2
PLs max, ng/mL	58.2	45.4
PLs range, ng/mL	51.3	39.2
% Patients > 20 ng/mL	75	63
% Patients < 20 ng/mL	16	18
% Patients < 10 ng/mL	1	18

Max, maximum value; Min, minimum value.

**FIGURE 6:**
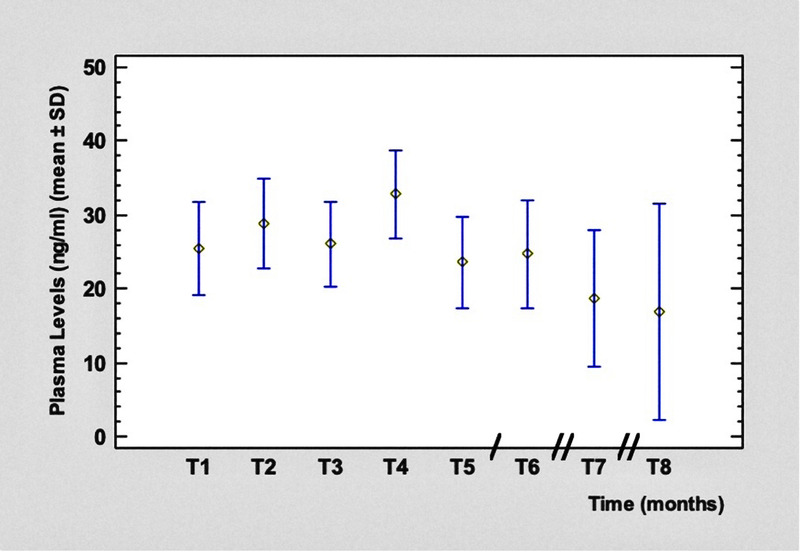
Time course of paliperidone PLs. Legend: / = 2 months; // = 3 months. Color image is available online only at www.psychopharmacology.com.

The time course of blood concentrations is shown in Figure 6. After an initial slight increase, after the fourth administration of PP1M, we observed a stabilization in PLs values, which tended to decrease slightly during treatment with PP3M. This reduction was not statistically significant.

## DISCUSSION

### Effectiveness

All patients treated first with PP1M and then with PP3M were previously stabilized with oral paliperidone until the acute episode resolution; therefore, our study investigated the possibility to avoid psychotic relapses, maintain clinical stabilization, and improve the general clinical condition. During the months on PP1M, patients showed an initial improvement in the psychopathological rating scales, at least from a statistical point of view, substantially maintaining a stable clinical picture during the many months on PP3M until the end of the observation period. In particular, the HOST subscale showed a significant reduction, including items like hostility, suspiciousness, and lack of cooperation, and this might be the effect of the maintenance of good compliance.

Other clusters of the BPRS scale tended to improve as well, and although the improvement was not statistically significant, it showed the consolidation of clinical stability. These results are in line with other literature data.^31–34^

Regarding the PANSS scale, general psychopathology and positive symptoms subscale scores showed a further clinical stabilization, emphasizing a good efficacy of paliperidone on positive symptoms, according to previous literature data on schizophrenic and schizoaffective patients.^20,24,35^

Similarly, Alphs et al^36^ (2014) evaluated the efficacy of PP1M in patients with schizophrenia, analyzing efficacy data by symptom domain of PANSS: symptom reduction regarded all major symptom domains, particularly that dose-dependent trends were observed for hostility and aggressive management.

We also observed an improvement of depressive symptoms during treatment with PP3M, evidenced by a significant reduction in HAM-D scores: this is interesting data if compared with the negative symptoms subscale trend, which was not significant, remarking the difference between affective core and negative symptoms, the latter being currently less responsive to antipsychotic treatment.^37,38^

Our results, with the exception of negative symptoms, were consistent with a 6-month study on patients with schizophrenia who were with PP1M after unsuccessful treatment with oral aripiprazole. Results showed relevant improvements in negative and depressive symptoms, conceptual disorganization, and global functioning.^39^

The study demonstrated that no other drug supplementation or dose adjustment was needed. Indeed, these preliminary data suggest that patients treated with PP1M/PP3M for 12 months tend to maintain clinical stability, in agreement with literature data.^33,35,40,41^

Only 1 of our patients experienced relapse and rehospitalization after the first injection of PP3M, showing low PLs (<20 ng/mL), probably due to an insufficient PP3M dosage for the severity of the clinical picture. Thus, we might consider hospitalization and discontinuation rates as a secondary outcome in long-term treatment, with low discontinuation rates as a predictive factor of better clinical outcome, considering the usual high rates of nonadherence to treatment by patients with schizophrenia. Our data confirm that long-acting therapy is one of the best options to reduce the number of hospitalizations and therapeutic failures due to poor compliance.

### Safety

Thirty-six percent of patients reported adverse effects, consistently with other literature data.^18,20^ No adverse effects were reported in 64% of patients at any time during the study. In particular, body weight showed a global variation of 4% and weight gain greater than 7% was reported in 5% of patients. In contrast, other articles showed a significant increase in body weight (>7%) in up to 42% of patients with schizophrenia treated with PP.^42^

Increased prolactin levels were found in 9%, dyskinesia in 5%, sexual dysfunction in 5%, and sedation in 14% during the first day after the injection. A review conducted by Mauri et al^20^ about the safety of short- and long-term paliperidone reported an incidence of extrapyramidal symptoms of 6% in long-term studies.

Hyperprolactinemia, as reported by many authors, was dose-dependent and higher in women^18^; an incidence of 9.5% was reported by a study on 21 schizophrenic and schizoaffective patients treated with PP,^20^ and a slightly higher incidence (13.9%) was reported in a review about PP in schizoaffective disorder^43^; sexual dysfunction was reported in 2% of cases.^42^ Pain at the injection site was reported in 9.6% of patients; injection site pain is reported by literature in less than 15% of cases.^18,20^

Regarding the metabolic profile, in our study, as in other previous ones, no significant changes were observed in laboratory tests (glycemia, total CHO, Try, GGT).^14,15,20,44^

Regarding dyskinesia, the incidence would probably cease to be relevant on a larger sample of patients, but it should be noted with concern, and clinicians must be aware that the risk of tardive dyskinesia is possible with second-generation antipsychotics.

### Plasma Levels

The results of our study confirmed the stability of PLs, after the steady state was reached through the PP1M injections, maintaining the stability of plasma concentrations of paliperidone with the trimestral formulation, especially in the transition phase between the 2 formulations, with no oral supplementation of the drug.

Nazirizadeh et al,^45^ in a multicenter retrospective analysis, examined the relationship between paliperidone serum concentrations and clinical effects in 217 patients who were followed by TDM; the mean concentration of paliperidone was 36 ± 25 ng/mL and the mean dose corrected concentration (C/D) was 4.7 ± 2.9 ng/mL per milligram. Among patients receiving paliperidone as antipsychotic monotherapy and who showed at least a much-improved level according to the Clinical Global Impression Scale scores, the 25th to 75th percentiles of paliperidone concentrations were 20 to 52 ng/mL. These results, in line with those of our study, were very similar to the AGNP (Arbeitsgemeinschaft für Neuropsychopharmakologie und Pharmakopsychiatrie) therapeutic reference range of 20 to 60 ng/mL for risperidone plus 9-hydroxy-risperidone (paliperidone, the active moiety). In the study by Nazirizadeh et al,^45^ the intraindividual and interindividual variabilities of trough serum concentrations were similar for paliperidone and risperidone active moiety, ranging between 30% and 35%.^46,47^ Although not significant, in our study, there was what looked like a clinically meaningful drop level—from a mean of 33 ng/mL to 24 ng/mL, which is a reduction of 27%. This reduction could mean that the conversion ratio for an equivalent dose of PP3M, when it replaces PP1M, needs to be re-evaluated. It also could mean that patients, when on PP1M, were administered more than they needed and could afford a dose reduction without reduction of effectiveness. This has been a common experience throughout the LAI literature, starting with fluphenazine and haloperidol decanoates—that patients develop lower levels when converted to the LAI and still do well, or even better than when on the oral formulation. We consider that PLs can drop during the phase of transition to PP3M, but they should stay greater than 20 ng/mL—an indicative value in view of the clinical efficacy showed by lower PLs in our sample population.

### Limitations

The present study has some limitations. First, in the small sample size, a larger number of patients should be involved to evaluate the clinical implications of formulation switch. The study is still ongoing; therefore, the presented data should be considered as preliminary, and a longer follow-up is recommended to confirm the clinical stability over time. A long-term follow-up might be advised to assess the impact on quality of life and global functioning of the extended dosing interval (4 doses per year). Moreover, patients could be differentiated on the basis of their response rates and their characteristics analyzed to optimize the treatment with long-acting paliperidone.

## CONCLUSIONS

The preliminary data of our study generally revealed a good long-term effectiveness of PP3M, with a tolerability profile consistent with that of PP1M. The absence of critical issues in the transition phase from PP1M to PP3M was confirmed by our results. Three-monthly paliperidone palmitate formulation showed to guarantee good therapeutic compliance and control of clinical symptomatology, thanks to constant plasma level maintenance. Currently, paliperidone palmitate is the only antipsychotic available in a PP3M in Italy and should be judged a helpful tool by clinicians for patients previously treated with PP1M to improve treatment adherence, offering advantages to patients with schizophrenia and their caregivers.

The limits of our study have been underlined considering the intraindividual and interindividual variability of PLP PLs as reported by other authors. We think that clinicians should be aware not only of the benefits but also of the low knowledge on the pharmacokinetic of PP3M, including the problems linked to the use of this “long long acting” formulation. It is a well-known fact that the range of drug PLs is extremely variable, and a relationship between drug PLs and clinical efficacy was rarely reported, being currently under discussion.
